# Elements of Inorganic Chemistry

**Published:** 1858-09

**Authors:** 


ART. VII.—*Elements of Inorganic Chemistry; including the Application of the Science in the Arts.* By THOMAS GRAHAM, F.R.S.L. and E. Late Professor of Chemistry in University College, London. Edited by HENRY WATTS, B.A., F.C.S., and ROBERT BRIDGES, M.D. Second American, from the second revised and enlarged London edition. 8vo., pp. 852. Philadelphia: Blanchard & Lea, 1858.

THE first portion of this work, comprising about one-half of its present contents, was edited by Dr. Bridges, in 1852, and at the time of its appearance was one of the most reliable guides to the student and teacher, and at once took a high position as a valuable standard production. The additions of the American editor were necessarily small, as the author had treated each subject in full, and had embodied in the work almost all the recent improvements and discoveries in the science. As the work now appears, it forms a complete repertory of all the facts and theories of the department of which it treats. Many changes and valuable additions have been made by the author and by Mr. Watts, the English editor, who has, in a supplement of nearly two hundred pages, so treated of all new facts and recent improvements as to render the first part of the work as full as the last. This supplement should have been inserted in the general text; a circumstance which would, in our opinion, have rendered it more complete. With the exception of some typographical errors, the volume is gotten up in a most unexceptionable manner; the type and binding are good, and the letter-press is amply illustrated by wood-cuts. We have no hesitation in recommending the work as being probably the most complete, in every respect, on Inorganic Chemistry in any language.

